# De Novo Assembly of Eight Commercial Crossbred Pig Genomes Provides Insights into the Potential Functional Impact of Structural Variation Hotspots

**DOI:** 10.3390/biom16020214

**Published:** 2026-01-31

**Authors:** Jiaolong Wen, Haiqi Qiu, Shaoxiong Deng, Shiyuan Wang, Yiyi Liu, Meng Lin, Jie Yang, Zhenfang Wu, Langqing Liu, Yibin Qiu

**Affiliations:** 1 National Engineering Research Center for Breeding Swine Industry, South China Agricultural University, Guangzhou 510642, China; dragon123@stu.scau.edu.cn (J.W.); haiqi_qiu@stu.scau.edu.cn (H.Q.); dengsx1998@stu.scau.edu.cn (S.D.); wsy20221024010@stu.scau.edu.cn (S.W.); yiyiliu@stu.scau.edu.cn (Y.L.); linmeng@scau.edu.cn (M.L.); jieyang@scau.edu.cn (J.Y.); wzf@scau.edu.cn (Z.W.); 2 Yunfu Subcenter of Guangdong Laboratory for Lingnan Modern Agriculture, Yunfu 527400, China; 3 National and Regional Livestock Genebank, Guangdong Gene Bank of Livestock and Poultry, South China Agricultural University, Guangzhou 510642, China; 4 Guangdong Provincial Key Laboratory of Agro-Animal Genomics and Molecular Breeding, South China Agricultural University, Guangzhou 510642, China

**Keywords:** haplotype-resolved, genome assembly, Duroc × (Landrace × Yorkshire) pig, comparative analysis

## Abstract

The Duroc × (Landrace × Yorkshire) (DLY) pig is a cornerstone of three-way crossbreeding system. Nevertheless, advances in commercial crossbred performance have been constrained by the dearth of high-resolution genomic resources for this key population. Here, we report the sequencing and assembly of 16 haplotype-resolved, chromosome-level genome assemblies derived from eight DLY pigs. These assemblies exhibited high continuity (contig N50: 18.17–29.54 Mb) and completeness (BUSCO: 99.3–99.4%), with sequences successfully localized to the 19 chromosomes. Genome annotation revealed an average of 21,922 protein-coding genes and 44.66% repetitive sequences per assembly. Comparative genomic analysis against the current reference genome Sscrofa11.1 enabled the construction of a non-redundant SV catalog comprising 130,416 variants, nearly half of which (48.99%) were novel relative to existing pig pan-genome SV panel. These SVs clustered non-randomly into 231 “SV hotspots” that were significantly enriched in protein-coding genes and putative regulatory elements. Functional analyses further linked these SV hotspots to quantitative trait loci (QTLs) associated with economically important traits. A focused analysis of a 3.43 Mb hotspot on chromosome 1, overlapping a known QTL for average daily gain, revealed eight high-frequency SVs in open chromatin regions near candidate genes (*NCS1*, *HMCN2*, *FUBP3*, *ABL1*, and *FIBCD1*), suggesting a cis-regulatory mechanism that may influence gene expression. Collectively, this work provides the first haplotype-resolved genomic resource for commercial crossbred pigs, and establishes a foundational framework for deciphering the genomic architecture of hybrid vigor and advancing precision breeding in swine.

## 1. Introduction

The pig is a critically important livestock species for meat production worldwide [1]. Modern commercial production primarily relies on a three-breed terminal crossing systems to optimize productivity, wherein F1 sows (Landrace × Large White) are bred with purebred Duroc boars selected for superior production traits such as growth rate, leanness, and feed efficiency [2,3]. The resulting hybrid offspring Duroc × (Landrace × Yorkshire), commonly termed DLY pigs, constitute a substantial portion of the meat supply, meeting growing consumer demand for high-quality protein [4].

Beyond their production value, DLY pigs offer a distinct advantage for the genetic dissection of economically important traits. Compared with purebred populations, their hybrid genomes exhibit shorter linkage disequilibrium (LD) due to the recombination of parental haplotypes [5]. This accelerated LD decay enables more precise mapping of quantitative trait loci (QTL), allowing finer resolution of genomic regions associated with key performance traits [6]. Consequently, DLY populations have been widely adopted in pig genetic studies [6,7,8,9].

Over the past decade, genomic and genetic research in pigs has largely relied on a single Duroc-origin reference genome [1,10]. While invaluable, a single reference poses limitations for comprehensively understanding genomic architecture, haplotype diversity, and the genetic basis of heterosis in hybrid breeding systems [11]. The absence of high-quality, haplotype-resolved reference genomes for widely used commercial hybrids like DLY constrains the full potential of genomic selection [12]. Thus, developing such resources is essential to advance the accuracy and efficiency of modern pig breeding programs.

Structural variants (SVs) are now recognized as key determinants of phenotypic diversity [13,14,15]. Recent research has highlighted the importance of population-level SV catalogs as critical resources for understanding genomic diversity and its functional implications [16,17,18]. However, the contribution of large-size genomic variants (≥50 bp), e.g., genome assembly dependent SVs, in shaping the genomic architecture of pigs remains poorly characterized. A major limiting factor is the scarcity of precise genetic variation information in hybrid lines. Of the 45 pig genomes currently available in NCBI database https://www.ncbi.nlm.nih.gov/datasets/genome/?taxon=9823 (accessed on 9 December 2025), only two (USMARCv1.0 and NCMD) represents crossbred animals [10,19].

To bridge these gaps, we sampled eight DLY pigs and successfully assembled high-quality, haplotype-resolved genomes by integrating Oxford Nanopore long-read and short-read sequencing data. We further identified genomic variants, with a focus on SVs, to construct a comprehensive variant catalog for this key commercial population and evaluated their potential functional contributions. This comprehensive dataset provides a valuable genetic resource that will enhance our understanding of the biological mechanisms underlying economically important traits and disease resilience in pigs.

## 2. Materials and Methods

### 2.1. Sample Collection

Ear tissue samples were collected from eight Duroc × (Landrace × Yorkshire) three-way crossbred (DLY) pigs (three males and five females) at 180–200 days of age. To avoid full- and half-sibling relationships, all individuals were selected based on a three-generation pedigree. The pigs were provided by the Wens Foodstuff Group Co., Ltd. (Yunfu, China). Samples were immediately snap-frozen in liquid nitrogen and stored at −80 °C until DNA extraction. All experimental protocols were approved by the Animal Care and Use Committee of the South China Agricultural University (approval number: SYXK 2019-0136, Guangzhou, China). No anesthesia or euthanasia was performed on the animals throughout this study.

### 2.2. Data Generation

Sequencing services were provided by Novogene Biotech Co., Ltd. (Beijing, China). Briefly, high-quality genomic DNA was isolated from the ear tissues (see Supplementary Method). For short-read sequencing, libraries were sequenced on a DNBSEQ-T7 platform, generating 150 bp paired-end reads. In total, 628.81 Gb of short-read data were produced, achieving a coverage ranging from 26.44× to 57.42× per individual (Table S1). To obtain long-read data, DNA libraries were prepared and sequenced on a Nanopore PromethION platform following standard Oxford Nanopore Technologies (ONT) protocols. Base-calling was performed with dorado (v1.3; https://github.com/nanoporetech/dorado (accessed on 9 December 2025)) using default parameters, retaining reads with an average quality score above 7. This yielded 565.44 Gb of ONT data with a mean read N50 of 19.28 kb and an average quality score of 12.89, providing 20.61× to 38.09× coverage across the eight samples (Table S1).

### 2.3. De Novo Genome Assembly

For each individual, sequence data were pre-processed before assembly. Short reads were trimmed and quality-controlled using Fastp [20] (v0.23.4). Corresponding ONT reads were error-corrected with Ratatosk [21] (v0.9.0) using the trimmed short reads as reference (Table S1). De novo genome assembly was performed per sample using the corrected ONT reads with Flye [22] (v2.9.6). The resulting draft assemblies were then polished into diploid contig-level sequences using Hypo-hybrid [23] (v1.0.3), which integrates both short and long-read data. Finally, chromosome-level scaffolding was carried out for each diploid assembly using the reference-guided tool RagTag [24] (v2.1.0), yielding two haplotype-resolved genomes per individual. Heterozygous regions between the two haplotypes were visualized with Bandage [25] (v0.9.0) by examining bubbles across the assembly graph of chromosomes (https://github.com/T2T-CN1/CN1/tree/main/heterozygosity (accessed on 9 December 2025)).

Assembly base quality (QV) was estimated using merqury [26] (v1.3), with k-mer databases constructed from short reads via meryl [26] (v1.4.1). Scaffold continuity was assessed with QUAST (v5.3.0; https://github.com/ablab/quast (accessed on 9 December 2025)). Assembly completeness was evaluated with Benchmarking Universal Single-Copy Orthologs (BUSCO) [27] (v6.1.0) against the mammalian single-copy ortholog set (mammalia_odb10) using the “--mode genome” option. Synteny between the newly assembled genomes and the reference genome (Sscrofa11.1) was analyzed using minimap2 [28] (v2.30) with “-asm 5” parameter and visualized with the pafr R package (v0.0.2; https://github.com/dwinter/pafr (accessed on 9 December 2025)).

### 2.4. Genome Annotation

Protein-coding genes were annotated by mapping the Sscrofa11.1 annotation file (Sus_scrofa.Sscrofa11.1.115.gff3) onto the assembled genomes using LiftOn [29] (v1.7.0). Transcript and protein sequences were extracted using gffread [30] (v0.12.7). Gene pairs located within collinearity blocks—identified from coding sequence alignments between the assembled genomes and the pig reference—were visualized in karyotype plots using JCVI [31] (v1.5.9). The completeness of the annotated transcriptomes and proteomes for each assembled genome was evaluated separately with BUSCO [27] (v6.1.0) using “--mode transcriptome” and “--mode proteins” options, respectively.

Repeat sequences were identified with a homology-based approach using RepeatMasker (v4.1.1; https://www.repeatmasker.org (accessed on 9 December 2025)). The RMBlast (v2.9.0; http://www.repeatmasker.org/rmblast/ (accessed on 9 December 2025)) search engine was employed with the transposable element (TE) databases from Dfam (v3.2; https://dfam.org (accessed on 9 December 2025)) and Repbase [32] (v20181026). The repeat landscape was visualized using the RepeatMasker utility scripts calcDivergenceFromAlign.pl and createRepeatLandscape.pl.

### 2.5. Structural Variant Calling

SVs were identified using two complementary approaches. First, ONT reads were aligned to the reference genome (Sscrofa11.1) using minimap2 [28] (v2.30) and SVs were called with Sniffles2 (v2.7.1; https://github.com/fritzsedlazeck/Sniffles (accessed on 9 December 2025)) under default parameters. Second, to detect SVs from genome assemblies, pairwise whole-genome alignments were performed with minimap2 [28] (v2.30) and SVs were called using syri [33] (v1.7.1) with default parameters. From both call sets, only variants labeled as “PASS” and located on autosomes were retained. Furthermore, deletions (DELs), duplications (DUPs), inversions (INVs), and insertions (INSs) larger than 50 bp were kept for downstream analysis. The SVs derived from the two methods were then merged using Truvari [34] (v5.4.0) with the “collapse” option to generate a non-redundant SV set. A Pig pan-genome SV panel [35] was downloaded (http://animal.omics.pro/code/index.php/panPig (accessed on 9 December 2025)) and compared against the non-redundant SVs set using Truvari [34] (v5.4.0) with the “bench” option.

### 2.6. Functional Annotation of SVs

Functional annotation of the identified SVs was performed using ANNOVAR [36]. SVs were categorized into seven groups based on genomic context: exonic and splicing (coding sequence variant), downstream (downstream gene variant), upstream (upstream gene variant), intronic (intron variant), intergenic (intergenic variant), UTR (UTR3 and UTR5), and others (ncRNA exonic, ncRNA intronic, and ncRNA splicing).

### 2.7. SVs Hotspot Identification

SV hotspots were identified using the “hotspotter” function from the primat R package (https://github.com/daewoooo/primatR (accessed on 9 December 2025)) with the parameters “bw = 200,000, pval = 1 × 10^−8^, num.trial = 2000”. To assess whether these SV hotspots were enriched in protein-coding genes and functional genomic regions. We extracted 22,018 unique protein-coding genes from Sscrofa11.1 annotation file (Sus_scrofa.Sscrofa11.1.115.gff3) and downloaded annotation of potential regulatory elements from Pan et al., 2021 [37]. Permutation tests for each feature set were carried out with the regioneR package (https://github.com/bernatgel/regioneR (accessed on 9 December 2025)) over 1000 iterations to evaluate statistical significance. For comparison, the same permutation tests were performed using all SVs as the background.

### 2.8. Functional Enrichment Analysis

Genes and quantitative trait loci (QTLs) that overlapped with SV hotspots were selected for functional enrichment analysis. Kyoto Encyclopedia of Genes and Genomes (KEGG) and Gene Ontology (GO) analyses were performed using KOBAS [38] (v3.0). For QTL enrichment analysis, QTL data were downloaded from PigBiobank (https://pigbiobank.piggtex.bio/download (accessed on 9 December 2025)), and enrichment was assessed using the GALLO R package [39]. Statistical significance was defined as an adjusted *p*-value < 0.05 based on the Benjamini–Hochberg value [40].

## 3. Results

### 3.1. High-Quality De Novo Assemblies for the DLY Pigs

**Figure 1:**
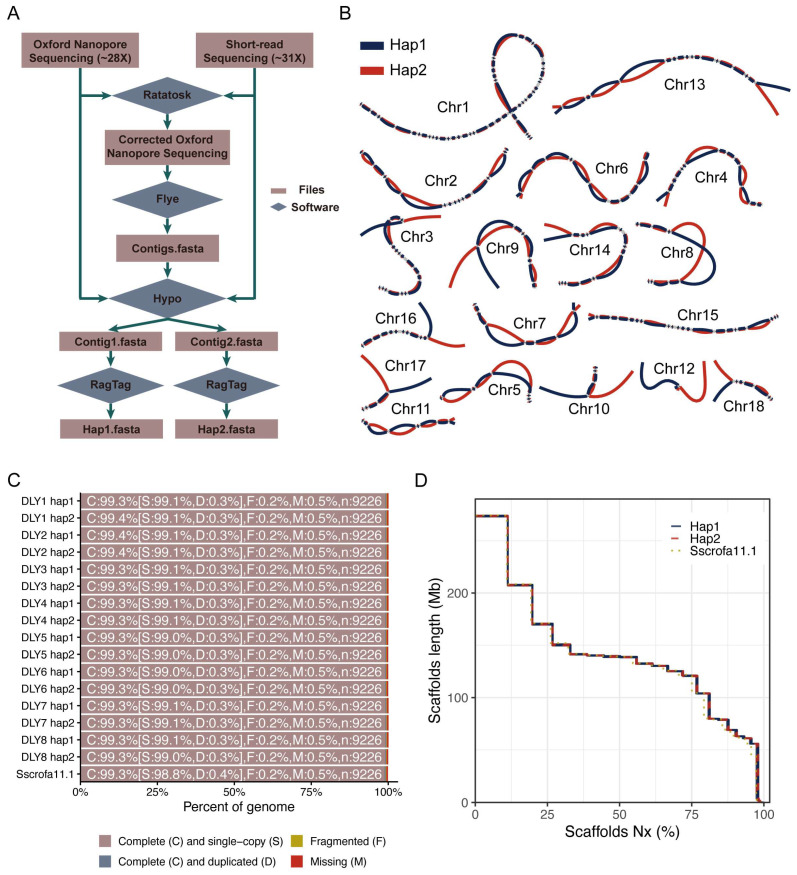
The genome assembly of the eight DLY pigs. (**A**) Workflow for the genome assembly of the eight DLY pigs. (**B**) Haplotype-resolved assemblies in the DLY pigs (using DLY2 as an example). Heterozygous regions between haplotype 1 (hap1) and haplotype 2 (hap2) in DLY2 are shown using bubbles across the assembly graphs of chromosomes (Chr). (**C**) BUSCO analysis of the DLY haplotype-resolved assemblies and the reference genome (Sscrofa11.1). (**D**) Cumulative lengths of the scaffolds in DLY haplotype-resolved assemblies and the reference genome (Sscrofa11.1). The x-axis indicates a scaffold Nx and the y-axis indicates the length of a scaffold Nx.

Using the combined long- and short-read sequencing data, we assembled draft genomes of the eight DLY pigs with Flye [22]. These assemblies had an average total length of 2.49 Gb, comprising 1787 contigs with a contig N50 of 24.36 Mb (Table S2). Subsequent phasing and chromosomal scaffolding yielded haplotype-resolved genome assemblies for each individual (Figure 1B). Relative to the pig reference genome (Sscrofa11.1), the haplotype-resolved assemblies ranged in size from 2.43 to 2.44 Gb, consisting of 687–1359 contigs with a contig N50 of 18.17–29.54 Mb and BUSCO completeness of 99.3–99.4% (Table 1; Figure 1C). Assembly base quality (QV) scores, estimated per individual with merqury using short-read data, averaged 43.02, exceeding that of the pig reference genome (QV = 36.48). Cumulative scaffold lengths of the 16 haplotype-resolved genomes demonstrated high assembly continuity (Table 1; Figure 1D). Moreover, the assembled sequences, ordered according to the reference genome, showed strong synteny with the reference (Figure S1).

### 3.2. Genome Annotation of the DLY Pigs

Repetitive elements were annotated across the 16 haplotype-resolved assemblies (Table S3; Figure S2). On average, 44.66% of each assembly was identified as repetitive sequence. Consistent with previous reports in pigs [19,41,42], LINEs constituted the most abundant repeat class (20.98% of each assembly), followed by SINEs (14.41%), LTRs (4.74%), and DNA transposons (2.45%).

Protein-coding gene annotation was performed by lifting over the reference annotation to the assemblies (Table 2). Between 21,846 and 22,001 genes (99.22–99.92%) were successfully transferred. The resulting annotations showed an average of 2.08 transcripts per gene, an average mRNA length of 59,939.94 bp, 11.7 exons per mRNA, and an average exon length of 269.31 bp. Completeness of the annotated transcriptomes and proteomes was assessed with BUSCO using the “mammalia_odb10” dataset (Table S4). For transcriptomes, an average of 96.32% of BUSCOs were complete, 1.42% fragmented, and 2.26% missing. For proteomes, 95.38% were complete, 1.96% fragmented, and 2.66% missing.

Furthermore, gene pairs located within collinearity blocks between each haplotype-resolved assembly and Sscrofa11.1 were compared, demonstrating a high degree of coding sequence conservation (Figure S3). Together, these annotation results confirm the high quality and functional completeness of the DLY pig genome assemblies.

### 3.3. Generating and Characterizing a Catalog of SVs in DLY Pigs

**Figure 2:**
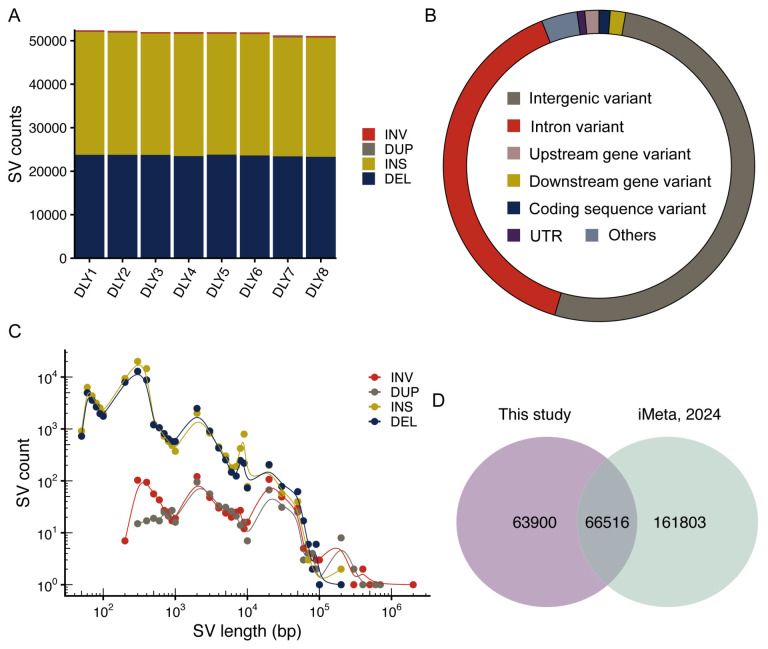
Characterization of SVs in DLY pigs. (**A**) The number of SV for each type per sample. (**B**) Functional annotation of SVs. (**C**) Length distribution of the SVs of each type. (**D**) Comparison analysis of the number of SVs.

SVs represent a major class of genetic variation [43], yet their detection in individual samples is often obscured by large-scale genomic synteny and collinearity (Figures S1 and S3). To comprehensively characterize SVs in the DLY pigs, we employed a combined alignment- and assembly-based detection strategy. On average, we detected 51,851 SVs (ranging from 51,094 to 52,409) in each genome, covering 34.06 Mb (ranging from 32.56 Mb to 35.83 Mb) (Figure 2A). Consequently, we merged the high-confidence SVs detected from all the samples, and constructed a set of 130,416 non-redundant SV catalog (length ≥ 50 bp), comprising 55,379 deletions, 73,500 insertions, 928 inversions, and 609 duplications (Table S5).

In-depth annotation revealed that the majority of SVs were located in intergenic (51.83%) or intronic regions (39.42%), while only a small fraction (1.15%) of SVs was found overlapped with protein-coding sequences (Figure 2B; Table S6). The size distribution of SVs showed distinct peaks corresponding to known transposable elements (Figure 2C). For example, two peak at lengths of ~55 bp and ~276 bp were mainly annotated as SINEs, a peak at ~1407 bp corresponds to LTR, and a peak at ~7964 bp corresponds to LINEs. This pattern is consistent with previous reports [15,44] and underscores the role of transposable elements as a major source of SVs in the pig genome.

We further compared our SV call set with a previously published pig pan-genome SV panel (Figure 2D) [35]. Notably, 48.99% of the SVs identified here were novel, highlighting the enhanced detection sensitivity afforded by long-read sequencing relative to assembly-based methods. This expanded SV catalog provides a valuable resource for future pan-genome and structural variation studies in pig.

### 3.4. Identification and Enrichment Analysis of SV Hotspots

**Figure 3:**
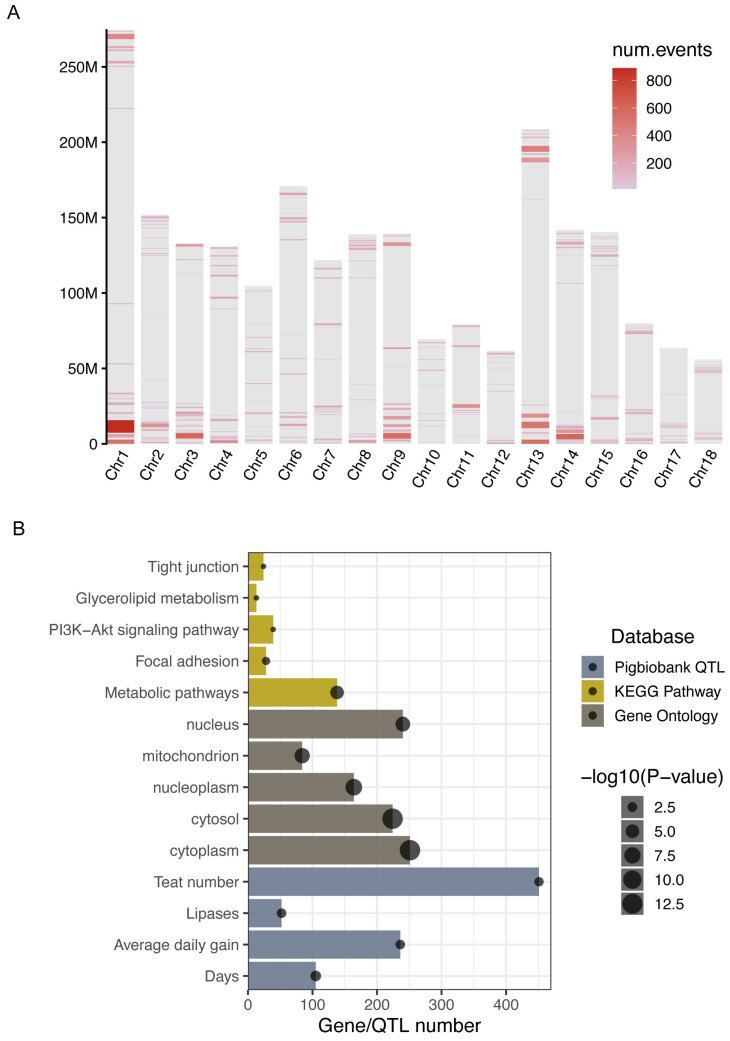
Enrichment analysis of SV hotspots. (**A**) Genome-wide distribution of SV hotspots. (**B**) KEGG, GO, and QTL enrichment analysis of SV hotspots. The detailed definitions for the enriched QTL terms are provided in Table S9.

The genomic SVs altered gene expression, and the resulting transcriptional changes effectively explained variation in heterosis, supporting the dominance model and highlighting a prevalent role of SVs in its genetic basis [11]. Although three-way crossbreeding systems are designed to utilize heterotic effects, the genome-wide landscape of structural variation in DLY pigs has not yet been systematically characterized. Here, we identified genomic regions enriched for SVs, hereafter referred to as “SV hotspots”, across the DLY pig genomes. Analysis revealed that SVs are non-randomly distributed [43,45], with 231 SV hotspots identified spanning approximately 203.69 Mb of the genome (Figure 3A; Table S7). To assess their functional relevance, we examined the overlap between these hotspots and annotated genomic features (Figure S4). SV hotspots showed significant enrichment in protein-coding genes (permutation test: *p* = 0.001, Z-score = 5.003). In contrast, when all SVs (non-redundant SVs) were tested against the same gene set, a significant depletion was observed (permutation test: *p* = 0.001, Z-score = −6.082). Similarly, comparison with putative regulatory elements [37] revealed significant overrepresentation of SV hotspots in these regions (permutation test: *p* = 0.002, Z-score = 3.145), whereas using all SVs as background showed significant depletion in regulatory elements (permutation test: *p* = 0.001, Z-score = −21.294).

A total of 2705 protein-coding genes and 4510 pig QTLs overlapped these SV hotspots. Functional enrichment analysis of the overlapping genes revealed several significantly enriched GO terms and KEGG pathways, including ATP binding, oxidation–reduction process, metabolic pathways, and fatty acid degradation (Figure 3B; Table S8). QTL enrichment analysis further indicated that these hotspots are implicated in growth-related traits such as days and average daily gain (Figure 3B; Table S9). As a representative example, a notable 3.43 Mb SV hotspot (Chr1:268,359,526–271,794,677) overlapped with a previously reported QTL (Chr1:270,153,237–271,111,196) associated with average daily gain (Table S10). Within this region, five candidate genes (*NCS1*, *HMCN2*, *FUBP3*, *ABL1*, and *FIBCD1*) were prioritized based on gene function and literature support. Further annotation identified eight high-frequency candidate SVs (present in ≥87.5% of DLY pigs) within this hotspot-QTL overlap that were enriched in open chromatin regions (Table S11), highlighting its possible role in modulating gene expression by altering the cis-regulatory elements that may contribute to heterosis in DLY pigs.

## 4. Discussion

Advances in long-read sequencing technologies and assembly algorithms have revolutionized genome assembly. Nevertheless, only 24 chromosome-level pig genomes are currently available in the NCBI database. In this study, we combined Oxford Nanopore long reads with short reads to generate 16 haplotype-resolved, chromosome-level genome assemblies that accurately capture the haplotype diversity of DLY commercial pigs. Our assemblies exhibit high accuracy, continuity, and completeness, and are expected to serve as an indispensable genomic resource for future studies. They will facilitate detailed haplotype comparisons, enhance the identification of heterozygous variants, and enable the assessment of genetic diversity at the individual level.

Although strong synteny and collinearity were observed between the newly assembled genomes and the pig reference genome (Sscrofa11.1), numerous SVs and sequence differences were also detected. The SVs identified in this study spanned approximately 98.94 Mb, representing about 3.95% of the pig reference genome. This SV profile differs from previously reported patterns of genomic variation [14,15], which can largely be attributed to the unique genetic background of DLY pigs. As a crossbred population derived from crossing F1 sows (Landrace × Large White) with purebred Duroc boars, a substantial proportion of the genomic variation in DLY pigs may originate from the Landrace and Large White lineages, as well as from recombination-derived SVs generated during hybridization. Additionally, the eight DLY pigs studied included three males and five females. Notably, we observed no substantial differences in SV profiles between sexes (Figure 2A), which is likely due to our analysis being confined to autosomes.

Consistent with other studies [14,15], we observed that SVs were enriched near chromosomal ends, likely because telomeres and subtelomeric regions are particularly prone to mutation [46]. Taking the SV hotspot region on chromosome 1 (Chr1:268.35–271.79 Mb) as an example, previous studies have reported a highly significant peak in this interval (Table S10). Alleles in several genes located here are linked to growth performance. For instance, *FUBP3* has been implicated in skeletal development and loin eye area [47,48], while *ABL1* is a reported candidate gene for backfat thickness [49] and meat-to-fat ratio in pigs [50]. Other genes in this region, such as *NCS1*, *HMCN2*, and *FIBCD1*, although not yet studied in pigs, have been associated with bone mineral density and body mass index in humans [51,52], suggesting potential conserved roles in growth regulation. Further investigation revealed eight high-frequency SVs located in open chromatin regions within this hotspot (Table S11). Given that presence/absence variants represent a major class of SVs and have been shown to play important roles in gene-expression heterosis [53], we speculate that these SVs may influence the expression of nearby genes by altering cis-regulatory elements, thereby contributing to phenotypic heterosis in pigs.

Lastly, it should be noted that scaffolding against Sscrofa11.1 in this study may have limited the detection of large-scale structural rearrangements. Furthermore, while chromosome-level continuity was achieved, gaps and potential misassemblies remain in highly complex genomic regions. Future efforts toward population-scale telomere-to-telomere assemblies will therefore be an important direction for refining structural variant discovery and genome completeness. We also observed variation in the number of putative coding genes annotated across haplotypes and individuals (Table 2). This variation likely reflects both biological differences, such as presence/absence variants that alter gene content (Table S12), and technical aspects of annotation transfer. Specifically, the accuracy of LiftOn is critically dependent on the accuracy of the source annotation, which may be less reliable for uncharacterized protein-coding genes. Consequently, the approach may fail to map such genes located in structurally complex or poorly aligned regions between the reference and the newly assembled genomes [29]. Indeed, many genes that were not successfully transferred are uncharacterized protein-coding genes, and a subset of these overlap with SV hotspots, assembly gaps, or unplaced contigs of the reference in our study (Table S12). These observations highlight that differences in gene counts across haplotypes and individuals result from a combination of genetic variation and limitations inherent to current annotation pipelines. Moving forward, integrating homology-based, RNA-seq-assisted, and ab initio gene predictions will be essential to achieve a more complete and accurate annotation of protein-coding genes in the pig genome. In addition, while we identified several candidate genes overlapping SVs, gene expression is often cell-, tissue-, stage-, or environment-specific [54,55]. The absence of parental genotype data and detailed growth phenotypes for the DLY pigs also limits our ability to directly correlate specific SVs with hybrid performance. Therefore, future studies incorporating multi-omics data, including transcriptomic, epigenomic, and phenotypic information, will be essential to functionally characterize these SVs and elucidate their roles in pigs.

## 5. Conclusions

Collectively, this study provides high-quality, haplotype-resolved, chromosome-level genome assemblies and constructs a comprehensive catalog of SVs for eight DLY commercial pigs. We demonstrate that SV hotspots are significantly enriched in protein-coding genes and potential regulatory elements, and highlight that high-frequency SVs within these regions likely contribute to economically important traits in commercial crossbred pigs. Our work establishes a foundational genomic resource that will support the fine-mapping of complex traits, facilitate haplotype-based selection, and advance the understanding of the genetic architecture underlying heterosis in pigs.

## Figures and Tables

**Table 1:** Genome statistics of the DLY haplotype-resolved assemblies and the reference genome (Sscrofa11.1).

ID	Assembly	Assembly Length (Gb)	Contig Number	Contig N50 (Mb)	Scaffold N50 (Mb)	Largest Scaffold Length (Mb)	Quality Values (QV)
DLY1	Hap1/Hap2	2.44/2.44	858/873	21.95/21.96	139/138.78	274.13/274.18	43.08/40.76
DLY2	Hap1/Hap2	2.43/2.43	796/791	29.54/29.54	138.76/138.79	274.14/274.10	45.87/42.89
DLY3	Hap1/Hap2	2.44/2.44	1359/1355	18.18/18.17	138.79/138.81	274.02/273.93	43.76/42.47
DLY4	Hap1/Hap2	2.44/2.44	810/805	28.14/28.15	138.89/138.92	273.19/273.21	46.09/43.03
DLY5	Hap1/Hap2	2.43/2.43	802/816	24.28/24.29	139.68/139.89	273.33/273.36	42.53/40.79
DLY6	Hap1/Hap2	2.43/2.43	855/857	25.12/25.12	138.85/138.87	273.26/273.35	46.56/43.12
DLY7	Hap1/Hap2	2.43/2.43	754/757	24.22/24.23	139.26/139.15	273.84/273.84	43.32/41.79
DLY8	Hap1/Hap2	2.43/2.43	690/687	22.67/22.68	139.14/139.16	273.85/273.89	42.27/40.04
Sscrofa11.1	Primary	2.5	1157	41.89	138.97	274.33	36.48

**Table 2:** Gene annotation statistics of the DLY haplotype-resolved assemblies and the reference genome (Sscrofa11.1).

ID	Assembly	Number of Putative Coding Genes	Number of mRNA	Average mRNA Length (bp)	Average CDS Length (bp)	Average Exons per mRNA	Average Exon Length (bp)
DLY1	Hap1/Hap2	21,930/21,910	45,688/45,652	59,961/59,833	1712/1695	11.7/11.7	269/269
DLY2	Hap1/Hap2	21,976/21,909	45,732/45,664	59,829/59,966	1705/1687	11.7/11.7	270/269
DLY3	Hap1/Hap2	21,923/21,867	45,663/45,607	59,832/60,018	1714/1711	11.7/11.7	269/269
DLY4	Hap1/Hap2	21,948/21,973	45,718/45,738	59,903/59,876	1715/1704	11.7/11.7	270/269
DLY5	Hap1/Hap2	22,001/21,970	45,740/45,709	59,872/59,925	1711/1699	11.7/11.7	270/270
DLY6	Hap1/Hap2	21,898/21,846	45,636/45,578	60,012/60,021	1713/1702	11.7/11.7	269/269
DLY7	Hap1/Hap2	21,934/21,887	45,692/45,641	59,940/60,036	1711/1700	11.7/11.7	270/269
DLY8	Hap1/Hap2	21,924/21,862	45,634/45,564	59,963/60,052	1692/1654	11.7/11.7	269/269
Sscrofa11.1	Primary	22,018	45,958	60,112	1732	11.8	268

## Data Availability

The assemblies of the 16 DLY haplotype-resolved, chromosome-level genomes can be obtained from https://doi.org/10.6084/m9.figshare.30951155 (accessed on 26 December 2025). Individual sequenced animals were proprietary properties of Guangdong Gene Bank of Livestock and Poultry. They may be requested by wzf@scau.edu.cn, respectively. Pig reference genome (Sscrofa11.1) and annotations (v115) can be obtained from ENSEMBL (https://ftp.ensembl.org/pub/release-115 (accessed on 26 December 2025)). QTL data were downloaded from https://pigbiobank.piggtex.bio/download (accessed on 26 December 2025) (PigBiobank_release1. The list of lead SNP in 300 studies.csv.gz). The workflow for the DLY pig genome assembly is available at https://github.com/YibinQiu/ONT_assembly_workflow (accessed on 26 December 2025).

## Supplementary Materials

The following supporting information can be downloaded at https://www.mdpi.com/article/10.3390/biom16020214/s1, Supplementary Method: Genomic DNA Extraction, Short-read Sequencing (DNBSEQ-T7), and Long-read Sequencing (Nanopore); Figure S1: Synteny analysis between the 16 DLY haplotype-resolved genomes and Sscrofa11.1; Figure S2: Sequence divergence of repetitive elements in the 16 DLY haplotype-resolved genomes; Figure S3: The alignment of coding sequences between 16 DLY haplotype-resolved genomes and Sscrofa11.1; Figure S4: The permutation result of annotated genomic features intersected with 231 SV hotspots; Table S1: The statistics of short- and long-reads sequencing sample; Table S2: The genome assembly statistics using Flye; Table S3: Repetitive elements across the 16 haplotype-resolved assemblies of the DLY pigs.; Table S4: Completeness of the annotated transcriptomes and proteomes; Table S5: Non-redundant SV catalog in DLY pigs; Table S6: Functional annotation of non-redundant SV catalog; Table S7: SV hotspots identified in this study; Table S8: GO and KEGG analysis using genes that overlapped with SV hotspots.; Table S9: QTL enrichment analysis using QTLs that overlapped with SV hotspots; Table S10: A 3.4 Mb SV hotspot overlapped with QTLs that associated with average daily gain and days; Table S11: The chromatin state overlapped with 14 candidate SVs; Table S12: The genes that failed to transfer from the reference genome annotation.

## Abbreviations

The following abbreviations are used in this manuscript:

DLY	Duroc × (Landrace × Yorkshire)
SV	Structural variant
LD	Linkage disequilibrium
QTL	Quantitative trait locus
ONT	Oxford Nanopore Technologies
KEGG	Kyoto Encyclopedia of Genes and Genomes
GO	Gene Ontology
