# Supplementary material for: De Novo Assembly of Eight Commercial Crossbred Pig Genomes Provides Insights into the Potential Functional Impact of Structural Variation Hotspots
Source: Biomolecules. 2026 Jan 31;16(2):214. doi: 10.3390/biom16020214 (PMC12938368; doi:10.3390/biom16020214)
Supplement: Supplementary file 1 [file biomolecules-16-00214-s001.zip › Supplementary Method and Figures S1-S4.pdf]

Supporting Information for

De Novo Assembly of Eight Commercial Crossbred Pig Genomes Provides Insights into the Potential Functional Impact of Structural Variation Hotspots

Jiaolong Wen [1,2,†], Haiqi Qiu [1,†], Shaoxiong Deng [1], Shiyuan Wang [1], Yiyi Liu [1], Meng Lin [1,3], Jie Yang [1,3], Zhenfang Wu [1,2,3], Langqing Liu [1,3,4*], Yibin Qiu [1,2,4*]

[1]National Engineering Research Center for Breeding Swine Industry, South China Agricultural University, Guangzhou, Guangdong, China

[2]Yunfu Subcenter of Guangdong Laboratory for Lingnan Modern Agriculture, Yunfu, Guangdong, China

[3]National and regional livestock genebank, Guangdong Gene Bank of Livestock and Poultry, South China Agricultural University, Guangzhou, Guangdong, China

[4]Guangdong Provincial Key Laboratory of Agro-animal Genomics and Molecular Breeding, South China Agricultural University, Guangzhou, Guangdong, China

*Corresponding authors: langqing.liu@scau.edu.cn; 13422157044qyb@gmail.com

†These authors contributed equally to this work

This word file includes:

Supplementary Method
Supplementary Figure S1 to S4
Legends for Supplementary Table S1 to S12

Supplementary Method

Genomic DNA Extraction

High quality of genomic DNA was isolated from ear tissue samples of pigs using an optimized SDS-based lysis protocol. Briefly, approximately 30–50 mg of tissue was homogenized in lysis buffer containing Proteinase K and SDS, followed by incubation at 56 °C for 2–4 h. After cooling to room temperature, the lysate was extracted with an equal volume of phenol/chloroform/isoamyl alcohol (25:24:1). DNA was precipitated with isopropyl alcohol, washed with 75 % ethanol, air-dried, and dissolved in TE buffer. DNA concentration was measured using a Qubit Fluorometer (Thermo Fisher Scientific), and the sample integrity and purity were assessed by agarose gel electrophoresis to ensure fragment sizes suitable for short-read sequencing.

Short-read Sequencing (DNBSEQ-T7)

Between 1–1.5 μg of genomic DNA was randomly fragmented by Covaris. Fragments of 200–400 bp were selected using the Agencourt AMPure XP-Medium kit (Beckman Coulter). Libraries were constructed through end-repair, adenylation, adapter ligation, and PCR amplification, followed by purification with the AxyPrep Mag PCR Clean-Up Kit. Single-strand circular DNA was generated using the MGIEasy Circularization Module (MGI) and quality-controlled before sequencing. Paired-end 150 bp sequencing was performed on the DNBSEQ-T7 platform, generating a total of 628.81 Gb of raw data.

Long-read Sequencing (Nanopore)

For Nanopore sequencing, we procured DNA from 500 μL of ear tissue using the Qiagen DNeasy kit in accordance with the manufacturer's guidelines. The DNA was eluted into 50 μL and subsequently concentrated to approximately 25 ng/μL using a Zymo DNA Clean and Concentrator Kit, resulting in a final elution volume of roughly 50 μL post-concentration. Fragment size was assessed by agarose gel electrophoresis to ensure fragment sizes suitable for long-read sequencing. Libraries were constructed using the SQK-LSK108 ligation kit (Oxford Nanopore Technologies) following the manufacturer's protocol. The final library was loaded onto a PromethION R9.4.1 flow cell and sequenced for 48 h using MinKNOW (v2.0).

Supplementary Figure S1 to S4

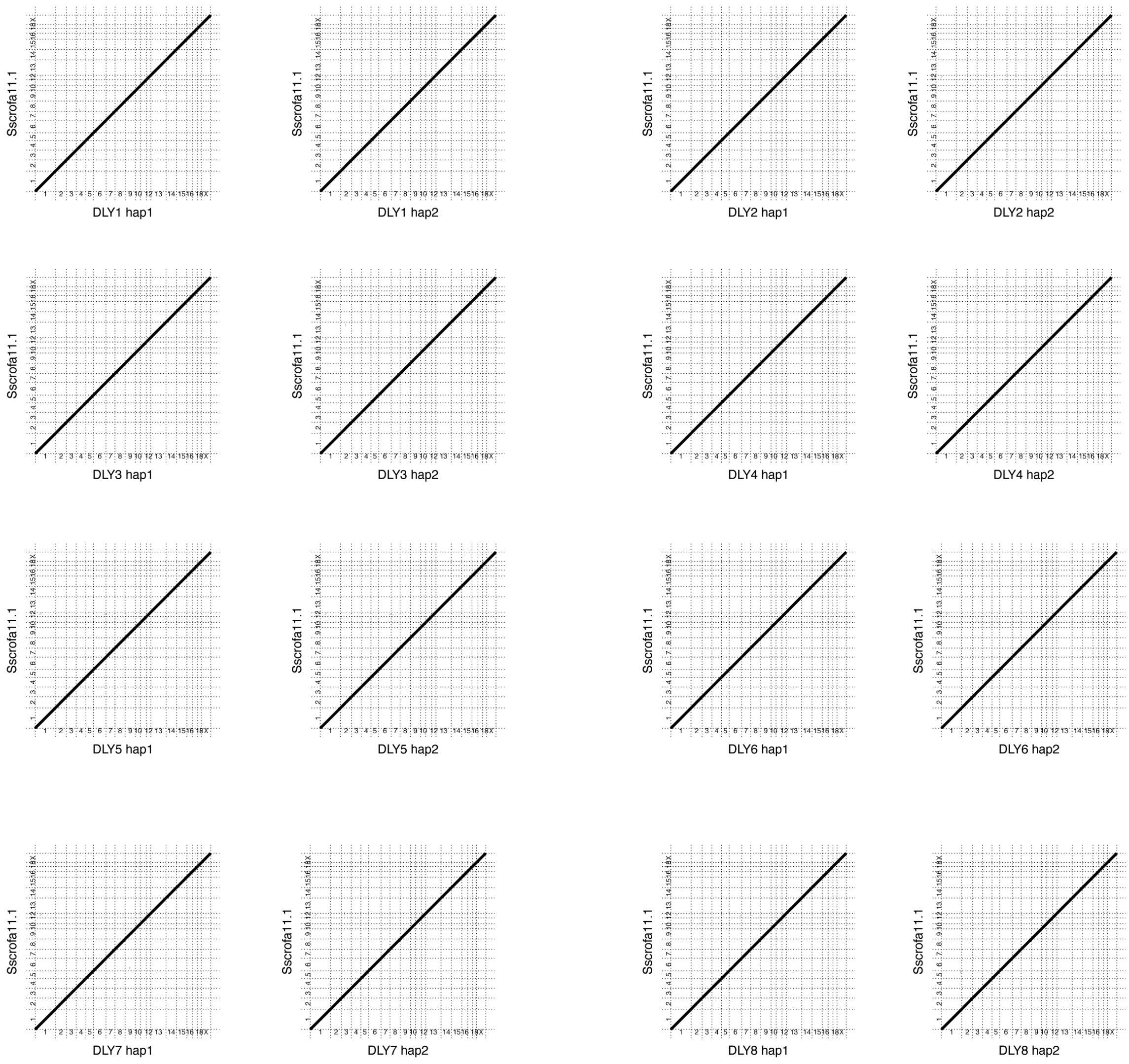

Figure S1. Synteny analysis between the 16 DLY haplotype-resolved genomes and Sscrofa11.1.

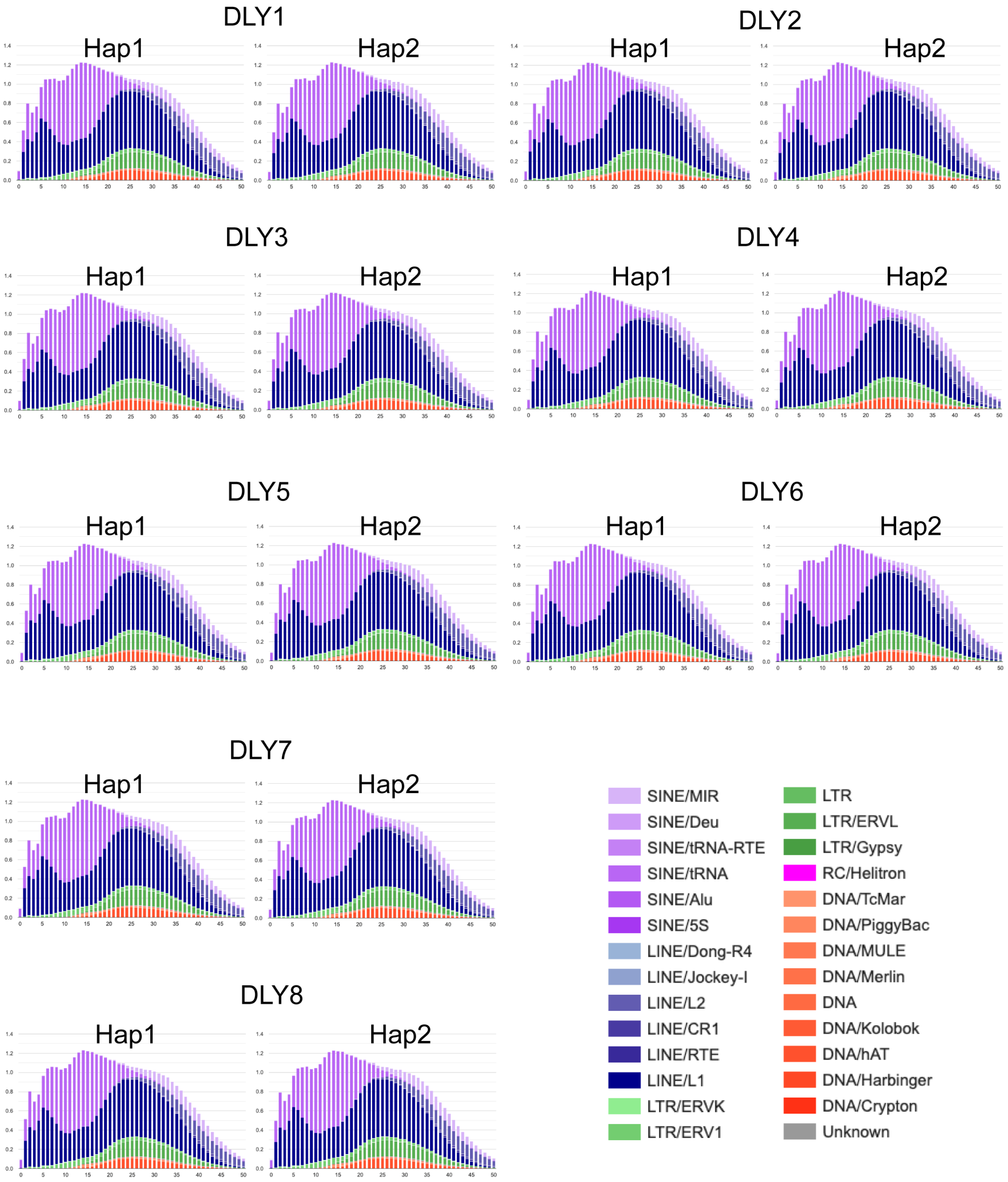

Figure S2. Sequence divergence of repetitive elements in the 16 DLY haplotype-resolved genomes.

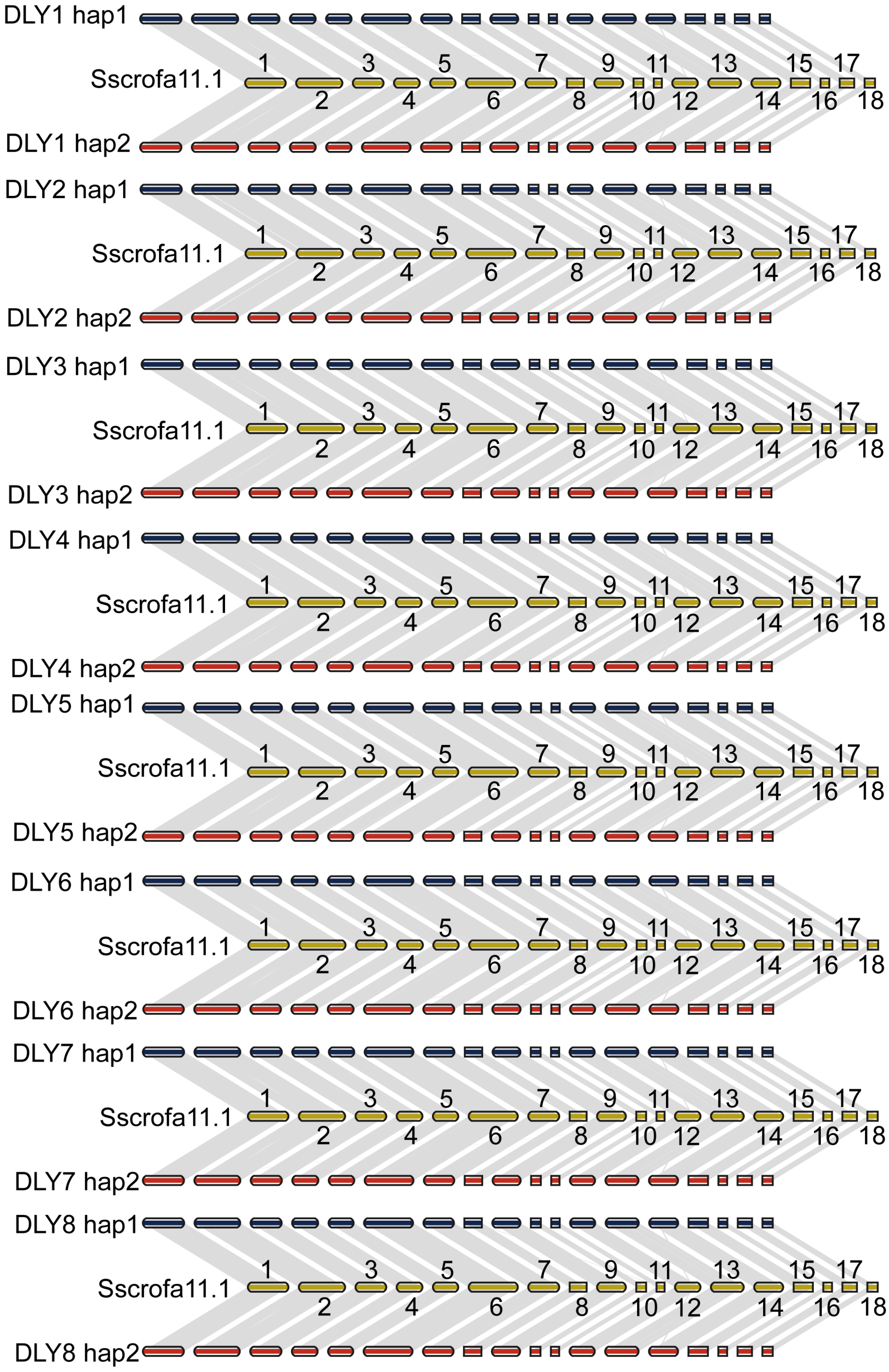

Figure S3. The alignment of coding sequences between 16 DLY haplotype-resolved genomes and Sscrofa11.1.

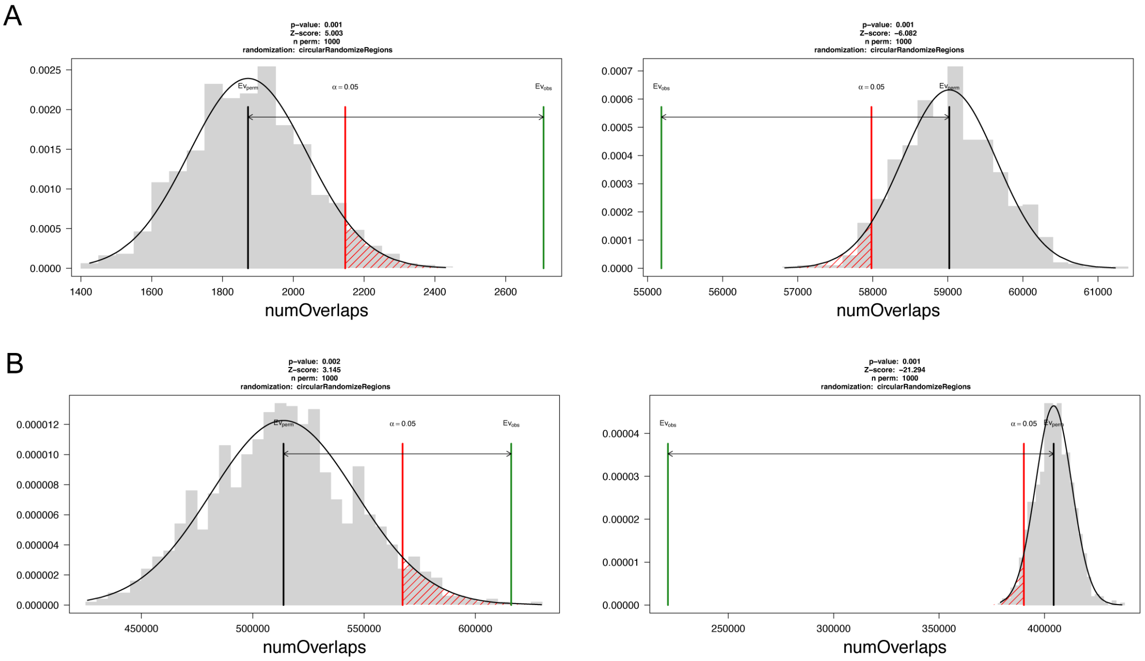

Figure S4. The permutation result of annotated genomic features intersected with 231 SV hotspots. (A) The permutation result of protein-coding genes intersected with 231 SV hotspots (left) and all SVs (right). (B) The permutation result of putative regulatory elements intersected with 231 SV hotspots (left) and all SVs (right). Gray regions represent the distribution of the evaluation of the randomized regions, the green line indicates the evaluation of the original region set, and the red line indicates the significance limit.

Legends for Supplementary Table S1 to S11

Supplementary Table S1 (separate file). The statistics of short- and long-reads sequencing sample.

Supplementary Table S2 (separate file). The genome assembly statistics using Flye.

Supplementary Table S3 (separate file). Repetitive elements across the 16 haplotype-resolved assemblies of the DLY pigs.

Supplementary Table S4 (separate file). Completeness of the annotated transcriptomes and proteomes.

Supplementary Table S5 (separate file). Non-redundant SV catalog in DLY pigs.

Supplementary Table S6 (separate file). Functional annotation of non-redundant SV catalog.

Supplementary Table S7 (separate file). SV hotspots identified in this study.

Supplementary Table S8 (separate file). GO and KEGG analysis using genes that overlapped with SV hotspots.

Supplementary Table S9 (separate file). QTL enrichment analysis using QTLs that overlapped with SV hotspots.

Supplementary Table S10 (separate file). A 3.4 Mb SV hotspot overlapped with QTLs that associated with average daily gain and days.

Supplementary Table S11 (separate file). The chromatin state overlapped with 14 candidate SVs.

Supplementary Table S12 (separate file). The genes that failed to transfer from the reference genome annotation.
